# A Prospective Study of Alcohol Consumption and Smoking and the Risk of Major Gastrointestinal Bleeding in Men

**DOI:** 10.1371/journal.pone.0165278

**Published:** 2016-11-08

**Authors:** Lisa L. Strate, Prashant Singh, Matthew R. Boylan, Sorbarikor Piawah, Yin Cao, Andrew T. Chan

**Affiliations:** 1 Department of Medicine, Division of Gastroenterology, University of Washington School of Medicine, Seattle, Washington, United States of America; 2 Department of Medicine, Massachusetts General Hospital, Harvard Medical School, Boston, Massachusetts, United States of America; 3 Department of Medicine, Division of Gastroenterology, Massachusetts General Hospital, Harvard Medical School, Boston, Massachusetts, United States of America; 4 Clinical and Translational Epidemiology Unit, Massachusetts General Hospital, Harvard Medical School, Boston, Massachusetts, United States of America; 5 Department of Medicine, Brigham and Women’s Hospital, Harvard Medical School, Boston, Massachusetts, United States of America; 6 Department of Nutrition, Harvard T.H. Chan School of Public Health, Boston, Massachusetts, United States of America; 7 Department of Medicine, Channing Division of Network Medicine, Brigham and Women’s Hospital, Harvard Medical School, Boston, Massachusetts, United States of America; University Hospital Llandough, UNITED KINGDOM

## Abstract

**Background and Aims:**

Data regarding smoking and alcohol consumption and risk of gastrointestinal bleeding (GIB) are sparse and conflicting. We assessed the risk of major GIB associated with smoking and alcohol consumption in a large, prospective cohort.

**Methods:**

We prospectively studied 48,000 men in the Health Professional follow-up Study (HPFS) who were aged 40–75 years at baseline in 1986. We identified men with major GIB requiring hospitalization and/or blood transfusion via biennial questionnaires and chart review.

**Results:**

We documented 305 episodes of major GIB during 26 years of follow-up. Men who consumed >30 g/day of alcohol had a multivariable relative risk (RR) of 1.43 (95% confidence interval (CI), 0.88–2.35; *P* for trend 0.006) for major GIB when compared with nondrinkers. Alcohol consumption appeared to be primarily related to upper GIB (multivariable RR for >30 g/day vs. nondrinkers was 1.35; 95% CI, 0.66–2.77; *P* for trend 0.02). Men who consumed ≥ 5 drinks/week vs. < 1 drink/month of liquor had a multivariable RR of 1.72 (95% CI, 1.26–2.35, *P* for trend <0.001). Wine and beer were not significantly associated with major GIB. The risk of GIB associated with NSAIDs/aspirin use increased with greater alcohol consumption (multivariable RR 1.37; 95% CI, 0.85–2.19 for 1-14g/day of alcohol, RR 1.75; 95% CI, 1.07–2.88 for ≥ 15g/day compared to nondrinkers). Smoking was not significantly associated with GIB.

**Conclusions:**

Alcohol consumption, but not smoking, was associated with an increased risk of major GIB. Associations were most notable for upper GIB associated with liquor intake. Alcohol appeared to potentiate the risk of NSAID-associated GIB.

## Introduction

Gastrointestinal bleeding (GIB) is a common and potentially life threatening medical problem that accounts for more than 200,000 inpatient admissions and 7,000 deaths in the United States each year.[1–3] Given the significant morbidity and mortality associated with GIB, it is important to identify potentially modifiable risk factors for GIB, such as smoking and alcohol consumption.

Alcohol consumption is a well-known risk factor for GIB associated with portal hypertension and cirrhosis. However, its association with other etiologies of major GIB is less clear. Most studies of non-variceal bleeding have focused on peptic ulcer. Some of these studies have found a positive association with alcohol consumption, [4,5] while others have failed to show an association.[6,7] Large population-based, prospective cohort studies of alcohol and the risk of overall GIB are currently lacking. In addition, data on the potential dose-response between alcohol and GIB are sparse. [4,8]

Existing studies of smoking and the risk of GIB are limited and conflicting.[5–18] Most investigations have been case-control or retrospective in design with limited data on smoking that may be prone to recall or selection bias. [6,7,9–11,13,14] In addition, existing studies have focused on the association of smoking with specific causes of GIB such as peptic ulcer [5,6,10] or diverticular bleeding [9,11,13] or in narrow populations such as patients in the peri-operative window [17] or with acute coronary syndromes. [18] Only a few population-based studies have examined the association of smoking with overall GIB.[19,20]

Therefore, we prospectively studied 48,000 men enrolled in the Health Professionals Follow-up Study (HPFS), a cohort study initiated in 1986 that we have previously used to establish that regular use of aspirin and/or NSAIDs is associated with a dose-dependent increase in risk of major GIB.[21] In the present study, we prospectively examine the association of alcohol and smoking, at a range of exposure, within the context of known or purported risk factors for GIB, including aspirin and NSAID use, and risk of major GIB over 20 years of follow-up.

## Methods

### Study Population

The HPFS is a prospective cohort of 51,529 male dentists, veterinarians, pharmacists, optometrists, osteopathic physicians and podiatrists who were aged 40 to 75 years at baseline in 1986 when they completed and returned a detailed medical and dietary questionnaire. Participants provide updated lifestyle and medical information biennially and dietary information every 4 years. This study was approved by the institutional review board at the Harvard T. H. Chan School of Public Health. Written consents were obtained from participants to review the medical records of those men who self- reported GIB.

### Ascertainment of Major GIB

**Fig 1:**
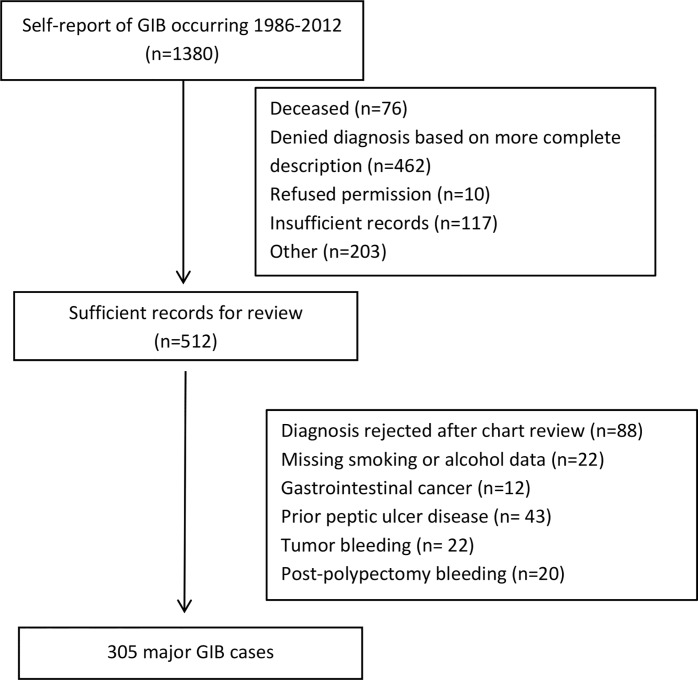
Study Flow Diagram. GIB, Gastrointestinal bleeding.

Starting in 2006, biennial questionnaires ascertained episodes of GIB that required hospitalization and/or blood transfusion. Participants reporting GIB were also asked to specify the site of bleeding (esophagus, stomach, duodenum, colon/rectum, other, unknown) and the year of diagnosis. A total of 1380 men self-reported a diagnosis of GIB occurring after 1986. Supplemental questionnaires were subsequently sent to these participants to assess further details regarding diagnosis and treatment, and to request permission to review medical records. A total of 462 patients were excluded based on the detailed information provided on the supplemental questionnaire. After further exclusions detailed in Fig 1, we obtained sufficient medical records for review in 512 participants. Two physicians blinded to exposure information, reviewed the records to validate self-reported cases and ascertain the etiology of bleeding. We defined major GIB as bleeding that required hospitalization and/or blood transfusion. Upper GIB was defined as bleeding originating from the esophagus, stomach, and duodenum, whereas lower GIB was defined as bleeding arising from the colon or rectum. A third reviewer resolved discrepancies in assigning etiology of bleeding. After excluding men who did not provide information on smoking status and alcohol intake, men with prior gastrointestinal cancer or peptic ulcer disease, and those with bleeding due to polypectomy or tumors, we rejected 88 cases of self-reported GIB and confirmed 305 cases of major GIB.

### Alcohol Consumption

Alcohol consumption was assessed every four years using a 131-item semi-quantitative food frequency questionnaire.[22] Participants were asked to report their average intake of beer, white wine, red wine, and liquor in the previous year according to nine possible response categories ranging from never or less than once per month to 6 or more times per day. We specified standard portions as a glass, bottle, or can of beer; a 4-oz glass of wine; and a shot of liquor. To determine total grams of alcohol intake, we multiplied the frequency of each beverage type by the average ethanol content in each portion (12.8 g per 12 oz. serving of beer; 11.3 g per 12 oz. of light beer; 11.0 g per 4 oz. of wine; 14.0 g per 1.5 oz. of liquor), and computed the sum of the beverage-specific intakes. In 2006, the portion size for wine was increased to 5 oz. and the alcohol content was adjusted accordingly. We also analyzed servings per day of each type of alcohol (e.g. wine, beer, liquor). For this analysis, we used servings rather than grams of alcohol in order to examine the effect of the type of drink rather than the absolute amount of alcohol. In 1994 and 1996, men were asked to report the proportion of alcohol consumed with meals. In a study of 136 men in the HPFS, there was excellent correlation between assessment of alcohol consumption by two food frequency questionnaires and two 1-week diet records over the same time period (Spearman correlation coefficient 0.86).[23]

### Smoking

Information on current smoking status and average number of cigarettes smoked per day was obtained at baseline and then updated biennially. Individuals were classified as nonsmokers, past smokers or current smokers. At baseline, past smokers were individuals who were not currently smoking but indicated having smoked 20 or more packs of cigarettes in their lifetime. During follow-up, individuals were classified as past smokers if they reported a prior history of smoking at least 20 packs of cigarettes and subsequently reported no smoking. We estimated smoking pack-years (equivalent of 20 cigarettes per day for one year) by multiplying the number of packs smoked per day with number of years smoked.

### Statistical Analysis

We excluded men who did not provide baseline information on smoking or alcohol consumption or who reported GI bleeding, peptic ulcer disease or gastrointestinal cancer prior to 1986. The remaining baseline population consisted of 48,000 men. We followed participants from the date of return of the baseline questionnaire in 1986 to the date of GIB, death, last questionnaire response, or the end of follow-up (December 31, 2012), and censored men at the time of diagnosis of gastrointestinal cancer or peptic ulcer disease without a report of GIB. We calculated age-specific incidence rates within 5-year age categories and used the Mantel Haenszel method to adjust for differences in the distribution of age groups among the exposure categories. We calculated age-adjusted and multivariable relative risks (RR) and 95% confidence intervals (CI) using Cox proportional hazards regression. Multivariable models were conditioned on age in 1-year intervals and 2-year study questionnaire cycle, and adjusted for body mass index (weight in kg/height in m^2^), physical activity (MET-h/week),[24,25] and regular use of aspirin (yes/no), regular use of NSAIDs (yes/no), and smoking or alcohol intake. Similar to prior analyses, alcohol consumption was modeled using updated cumulative intake to best estimate long-term exposure, and was grouped into the following categories: 0, 1–4, 5–14, 15–29, and ≥30g/day [26–28]. Using cumulative averaging, men who reported consuming no alcohol or less than one serving per month were considered long-term non-drinkers. Smoking status and pack-years were modeled using the most recent data reported on study questionnaires prior to the episode of GIB (simple updating). We grouped pack-years of smoking into five categories: 0, <10, 10–24, 25–44, and > = 45 pack-years. Covariates in the adjusted models such as NSAID use were modeled using simple updating. Categories were created for missing covariate data when appropriate. To test for linear trend between exposure amount and risk of GIB, we used the median value in each category of exposure as a continuous variable in the regression models.

For the analyses, we used SAS, version 9.3 (SAS Institute, Cary, North Carolina). All *P* values are two-sided, and *P* <0.05 was considered statistically significant.

## Results

During 883,797 person-years of follow-up, we documented 305 cases of major GIB including 142 cases of upper GIB and 126 cases of lower GIB. Age-standardized baseline characteristics of the study population are summarized in Table 1 according to smoking status and alcohol intake. In general, men who consumed more alcohol tended to be more physically active, more likely to be a current or past smoker and more likely to be regular users of aspirin or NSAIDs. Men who were past or current smokers tended to use more NSAIDs and aspirin and to consume more alcohol than never smokers. Current smokers were also less physically active than past or never smokers (Table 1).

**Table 1:** Baseline Characteristics of Study Cohort in 1986 According to Smoking Status and Alcohol Consumption.

	Smoking status			Alcohol Consumption (g/day)				
	Never	Past	Current	0	1–4	5–14	15–29	≥30
	(n = 21627)	(n = 19987)	(n = 4517)	(n = 10569)	(n = 10954)	(n = 12398)	(n = 6012)	(n = 5333)
Age, years	52.7(9.8)	55.7(9.7)	53.8(9.2)	54.2(9.9)	53.2(9.9)	53.6(9.6)	53.7(9.4)	55.3(9.5)
Body mass index, kg/m^2^	25.3(3.3)	25.8(3.3)	25.4(3.2)	25.7(3.6)	25.6(3.3)	25.4(3.1)	25.3(3.0)	25.5(3.2)
Physical activity, MET h/wk	21.7(29.4)	21.2(29.6)	15.3(23.1)	18.8(27.0)	20.2(26.7)	22.6(30.2)	24.0(34.5)	21.1(29.5)
NSAID use (%)^a^	8.2	11.1	11.1	8.5	9.1	9.7	11.3	12.4
Aspirin use (%)^a^	27.5	33.3	31.6	26.6	26.6	30.3	32.5	34.3
Cardiovascular disease (%)	3.4	5.9	4.8	4.9	4.9	4.3	3.7	4.1
Osteoarthritis (%)	8.3	9.3	8.9	8.5	8.2	8.4	8.8	9.9
Alcohol, g/day	8.0(12.2)	13.6(16.4)	17.5(20.4)					
Smoking status (%)								
Never				61.4	52.6	44.2	36.8	25.3
Past				31.7	39.7	46.7	53.5	54.2
Current				6.9	7.6	9.1	9.7	20.6

NOTE: All values are means (SD) or percentages. All variables except age are standardized to the age distribution of the study population. MET, metabolic equivalent; NSAID, nonsteroidal anti-inflammatory drug.

^a^ NSAID and aspirin use defined as regular use at least 2 times per week

### GIB and Alcohol Intake

We observed a linear association between the amount of alcohol intake and the risk of GIB. After controlling for other potential risk factors, individuals who consumed 30 or more g/day of alcohol had a multivariable relative risk (RR) of 1.43 (95% CI, 0.88–2.35; *P* trend 0.006) when compared with men who did not consume alcohol (Table 2). The effect of alcohol on GIB appeared to be primarily related to upper GIB. The multivariable RR for upper GIB was 1.35 (95% CI, 0.66–2.77, p for trend 0.02) in a highest to lowest comparison, whereas for lower GIB the RR was 1.18 (95% CI, 0.53–2.62, *P* trend 0.65). The risk of overall GIB and upper GIB was significantly elevated even with moderate alcohol consumption (15–29 g/day or 1–2 drinks/day; multivariable RR1.67; 95% CI, 1.09–2.55 and 1.76; 95% CI, 0.96–3.22, respectively, when compared to non-drinkers). When we excluded non-drinkers from the analysis, the results were similar. For example, compared to men who drank 1-4g/week, men who drank at least 30 g/week had a RR of 1.73 (95% CI, 0.57–5.19).

**Table 2:** Alcohol Consumption and Risk of Major GI Bleeding.

	Alcohol intake (g/day)					
	0	1–4	5–14	15–29	≥30	P value for linear trend^a^
Person-years	151156	244945	260621	139467	87607	
**All GI bleeding**^**b**^						
No. of cases	32	73	94	72	34	
Age-adjusted HR (95% CI)^c^	1.0	1.15 (0.76, 1.75)	1.35 (0.91, 2.02)	1.78 (1.17, 2.70)	1.56 (0.96, 2.54)	0.002
Multivariable1 HR (95% CI)^d^	1.0	1.10 (0.72, 1.67)	1.29 (0.86, 1.94)	1.67 (1.09, 2.55)	1.43 (0.88, 2.35)	0.006
Multivariable 2 HR (95% CI)^e^	1.0	1.08 (0.71,1.64)	1.27 (0.84,1.91)	1.64 (1.07,2.52)	1.41 (0.86,2.31)	0.007
**Upper GI bleeding**^**f**^						
No. of cases	16	31	44	36	15	
Age-adjusted HR (95% CI)^c^	1.0	0.98 (0.54, 1.79)	1.28 (0.72, 2.28)	1.79 (0.99, 3.23)	1.38 (0.68, 2.79)	0.01
Multivariable 1 HR (95% CI)^d^	1.0	0.95 (0.52, 1.73)	1.27 (0.71, 2.27)	1.76 (0.96, 3.22)	1.35 (0.66, 2.77)	0.02
Multivariable 2 HR (95% CI)^e^	1.0	0.93 (0.51, 1.71)	1.27 (0.71, 2.27)	1.77 (0.97, 3.24)	1.37 (0.67, 2.82)	0.01
**Lower GI bleeding**^**f**^						
No. of cases	13	36	39	26	12	
Age-adjusted HR (95% CI)^c^	1.0	1.41 (0.75, 2.67)	1.38 (0.74, 2.59)	1.58 (0.81, 3.08)	1.36 (0.62, 2.98)	0.42
Multivariable 1 HR (95% CI)^d^	1.0	1.33 (0.71, 2.52)	1.29 (0.68, 2.43)	1.45 (0.73, 2.85)	1.18 (0.53, 2.62)	0.65
Multivariable 2 HR (95% CI)^e^	1.0	1.27 (0.67, 2.40)	1.25 (0.66, 2.36)	1.38 (0.70, 2.73)	1.13 (0.51, 2.51)	0.69

^a^ *P* value for trend calculated using the median value in each category of alcohol consumption as a continuous variable in the regression models

^b^ Includes 28 cases of bleeding of unknown etiology and 9 cases of small bowel bleeding

^c^ Adjusted for age in years and study period in 4-year intervals; using cumulative updating to examine alcohol consumption

^d^ Adjusted for smoking (past/current), body mass index (<21, 25–29, 30–31, ≥32 kg/m^2^), physical activity (quintiles), regular use of aspirin (at least 2 times per week; yes/no), regular use of NSAIDs (at least 2 times per week; yes/no); using cumulative updating to examine alcohol consumption

^e^ Adjusted for multivariate model 1 plus medication use (proton pump inhibitors, H2 receptor antagonists, selective serotonin reuptake inhibitors, warfarin and/or clopidogrel) and comorbid disease (myocardial infarction, stroke or transient ischemic attack, rheumatoid arthritis, degenerative joint disease, peripheral vascular disease, chronic obstructive pulmonary disease, chronic kidney disease).

^f^ Upper GI bleeding was defined as bleeding originating from the esophagus, stomach, or duodenum; lower GI bleeding was defined as bleeding arising from the colon or rectum.

In an analysis of types of alcohol consumed, risk of GIB primarily appeared to increase linearly with amount of liquor consumed (Table 3). After adjusting for other potential risk factors, individuals who consumed 5 or more drinks/week of liquor had an increased risk of GIB (RR 1.72; 95% CI, 1.26–2.35, *P* trend <0.001) compared to those who consumed liquor less than once per month. This association also appeared to be mainly driven by upper GIB (multivariable RR 1.79; 95% CI, 1.14–2.82, *P* trend = 0.007 in a highest to lowest category comparison). When the analyses were further adjusted for total alcohol consumption, the results were not materially changed. The multivariable RR for men who consumed liquor 5 or more times per week compared to those who consumed < 1/month was 1.65 (95% CI, 1.06–2.56). Similarly, liquor remained significantly associated with GIB when we adjusted the models for all alcohol types. In high to low comparisons, the multivariable RR was 1.68 (95% CI, 1.20–2.36) for overall GIB and 1.69 (95% CI, 1.03–2.78) for upper GIB. Consumption of wine and beer (Table 3) did not appear to be associated with increased risk of GIB. The risks of GIB for red and white wine were similar. The correlations between consumption of the different types of alcohol (beer, wine and liquor) were weak (r < 0.1).

**Table 3:** Beverage-specific Alcohol Consumption and Risk of Major GI Bleeding.

	Alcohol Consumption (drinks)				
	< 1/month	1-4/month	2-4/week	≥5/week	P value for linear trend^a^
	**Liquor**				
Person-years	398395	214670	143354	123950	
**All GI bleeding**^**b**^					
No. of cases	111	63	61	70	
Age-adjusted HR (95% CI)^c^	1.0	1.11 (0.81, 1.51)	1.34 (0.98, 1.83)	1.86 (1.38, 2.51)	<0.001
Multivariable 1 HR (95% CI)^d^	1.0	1.07 (0.78, 1.46)	1.27 (0.92, 1.74)	1.72 (1.26, 2.35)	<0.001
Multivariable 2 HR (95% CI)^e^	1.0	1.06 (0.78,1.45)	1.26 (0.92,1.73)	1.70 (1.24,2.32)	0.002
**Upper GI bleeding**^**f**^					
No. of cases	53	29	28	32	
Age-adjusted HR (95% CI)^c^	1.0	1.06 (0.68, 1.67)	1.30 (0.82, 2.06)	1.80 (1.16, 2.81)	0.005
Multivariable 1 HR (95% CI)^d^	1.0	1.05 (0.67, 1.66)	1.28 (0.80, 2.03)	1.79 (1.14, 2.82)	0.007
Multivariable 2 HR (95% CI)^e^	1.0	1.05 (0.67,1.66)	1.28 (0.81, 2.04)	1.79 (1.13, 2.82)	0.007
**Lower GI bleeding**^**f**^					
No. of cases	50	27	25	24	
Age-adjusted HR (95% CI)^c^	1.0	1.06 (0.66, 1.70)	1.20 (0.74, 1.95)	1.39 (0.85, 2.27)	0.17
Multivariable 1 HR (95% CI)^d^	1.0	1.01 (0.63, 1.61)	1.12 (0.69, 1.82)	1.22 (0.74, 2.01)	0.39
Multivariable 2 HR (95% CI)^e^	1.0	0.99 (0.62, 1.59)	1.08 (0.66, 1.76)	1.17 (0.71, 1.94)	0.48
	**Wine**				
Person-years	278775	279441	207683	114886	
**All GI bleeding**^**b**^					
No. of cases	75	105	78	47	
Age-adjusted HR (95% CI)^c^	1.0	1.33 (0.99, 1.79)	1.12 (0.82, 1.54)	1.22 (0.84, 1.75)	0.91
Multivariable 1 HR (95% CI)^d^	1.0	1.29 (0.96, 1.74)	1.07 (0.78, 1.48)	1.18 (0.81, 1.71)	0.82
Multivariable 2 HR (95% CI)^e^	1.0	1.30 (0.96,1.75)	1.08 (0.78,1.48)	1.19 (0.82,1.73)	0.83
**Upper GI bleeding**^**f**^					
No. of cases	34	52	32	24	
Age-adjusted HR (95% CI)^c^	1.0	1.06 (0.72, 1.55)	1.13 (0.71, 1.79)	0.94 (0.40, 2.21)	0.93
Multivariable 1 HR (95% CI)^d^	1.0	1.44 (0.93, 2.22)	1.01 (0.63, 1.65)	1.37 (0.81, 2.31)	0.67
Multivariable 2 HR (95% CI) ^e^	1.0	1.42 (0.92, 2.20)	0.98 (0.60, 1.61)	1.38 (0.81, 2.34)	0.61
**Lower GI bleeding**^**f**^					
No. of cases	33	39	35	19	
Age-adjusted HR (95% CI)^c^	1.0	1.13 (0.71, 1.80)	1.14 (0.71, 1.84)	1.12 (0.64, 1.98)	0.94
Multivariable 1 HR (95% CI)^d^	1.0	1.09 (0.69, 1.74)	1.10 (0.68, 1.79)	1.09 (0.62, 1.94)	0.83
Multivariable 2 HR (95% CI)^e^	1.0	1.10 (0.69, 1.75)	1.09 (0.67, 1.76)	1.08 (0.61, 1.92)	0.87
	**Beer**				
Person-years	326872	266097	183822	102318	
**All GI bleeding**^**b**^					
No. of cases	97	92	82	34	
Age-adjusted HR (95% CI)^c^	1.0	1.13 (0.85, 1.50)	1.41 (1.05, 1.89)	1.22 (0.82, 1.81)	0.21
Multivariable 1 HR (95% CI)^d^	1.0	1.09 (0.82, 1.46)	1.35 (1.00, 1.82)	1.20 (0.81, 1.79)	0.25
Multivariable 2 HR (95% CI) ^e^	1.0	1.08 (0.81,1.44)	1.35 (1.00,1.82)	1.22 (0.82,1.81)	0.25
**Upper GI bleeding**^**f**^					
No. of cases	44	40	41	17	
Age-adjusted HR (95% CI)^c^	1.0	1.07 (0.70, 1.65)	1.53 (1.00, 2.35)	1.33 (0.76, 2.33)	0.18
Multivariable 1 HR (95% CI)^d^	1.0	1.06 (0.69, 1.63)	1.51 (0.98, 2.33)	1.35 (0.76, 2.39)	0.17
Multivariable 2 HR (95% CI)^e^	1.0	1.06 (0.68, 1.67)	1.30 (0.82, 2.06)	1.80 (1.16, 2.81)	0.15
**Lower GI bleeding**^**f**^					
No. of cases	39	44	30	13	
Age-adjusted HR (95% CI)^c^	1.0	1.35 (0.88, 2.08)	1.30 (0.81, 2.10)	1.18 (0.63, 2.21)	0.79
Multivariable 1 HR (95% CI)^d^	1.0	1.29 (0.84, 2.00)	1.24 (0.76, 2.01)	1.14 (0.60, 2.16)	0.88
Multivariable 2 HR (95% CI)^e^	1.0	1.27 (0.82, 1.96)	1.22 (0.75, 1.99)	1.16 (0.61, 2.19)	0.82

^a^ *P* value for trend calculated using the median value in each category of alcohol consumption as a continuous variable in the regression models

^b^ Includes 28 cases of bleeding of unknown etiology and 9 cases of small bowel bleeding

^c^ Adjusted for age in years and study period in 4-year intervals; using cumulative updating to examine alcohol consumption

^d^ Adjusted for smoking (past/current), body mass index (<21, 25–29, 30–31, ≥32 kg/m^2^), physical activity (quintiles), regular use of aspirin (at least 2 times per week; yes/no), regular use of NSAIDs (at least 2 times per week; yes/no); using cumulative updating to examine alcohol consumption

^e^ Adjusted for multivariate model 1 plus medication use (proton pump inhibitors, H2 receptor antagonists, selective serotonin reuptake inhibitors, warfarin and/or clopidogrel) and comorbid disease (myocardial infarction, stroke or transient ischemic attack, rheumatoid arthritis, degenerative joint disease, peripheral vascular disease, chronic obstructive pulmonary disease, chronic kidney disease).

^f^Upper GI bleeding was defined as bleeding originating from the esophagus, stomach, or duodenum; lower GI bleeding was defined as bleeding arising from the colon or rectum.

We examined whether the risk of GIB associated with NSAIDs/aspirin use was different among men who consumed alcohol. The risk of GIB among regular NSAID and/or aspirin users (at least twice per week)[29] increased with greater amounts of alcohol consumption (S1 Table). For overall GIB, the multivariable RR in NSAID/aspirin users were 1.37 (95% CI, 0.85–2.19) and 1.75 (95% CI, 1.07–2.88), and for non NSAID/aspirin users were 0.86 (95% CI, 0.43–1.73) and 1.44 (95% CI, 0.68–3.06) for men who consumed 1–14 g/day and ≥ 15g/day of alcohol, respectively, compared to nondrinkers.

We also examined drinking alcohol with and without meals to explore potential reasons for the relationship between consumption of liquor and risk of GIB. We found that the risk of GIB, particularly upper GIB, was more evident among men who consumed alcohol without meals compared to those who consumed with meals. In men who consumed >15 g/day of alcohol, the multivariable RR for upper GIB was 1.67 (95% CI, 0.99–2.81) in those who consumed <50% of their alcohol with meals, and 1.34 (95% CI, 0.69–2.59) in those who consumed >50% of their alcohol with meals when compared to nondrinkers (*P* interaction 0.09).

In an analysis of bleeding etiology, consumption of >15 g/day of alcohol was significantly associated with bleeding from peptic ulcer disease (multivariable RR 1.61; 95% CI, 0.82–3.14; *P* trend 0.03), but not esophagitis/gastritis/duodenitis or diverticular bleeding (S2 Table). Bleeding due to portal hypertension was rare (n = 4), and is unlikely to have influenced our results. Liquor and beer consumption of ≥5 drinks/day were associated with increased risk of bleeding from peptic ulcer disease (multivariable RR 1.61; 95% CI, 0.93–2.79; *P* trend 0.07 and multivariate RR 1.92; 95% CI, 1.05–3.51; *P* trend 0.02).

In a subanalysis conducted between 2008 and 2012, we were also able to control for use of proton pump inhibitors, H2 receptor antagonists, selective serotonin reuptake inhibitors, clopidogrel and warfarin. In addition, we adjusted for comorbid disease (osteoarthritis and rheumatoid arthritis, diabetes, myocardial infarction, stroke, peripheral vascular disease, chronic obstructive pulmonary disease, and chronic kidney disease) (Tables 2 and 3). In these analyses our results were not materially different. For example, in the fully adjusted model the RR was 1.41 (95% CI, 0.86–2.31) for men who consumed >30 g/ day compared to non-drinkers (*P* = 0.007).

Lastly, we performed an analysis including men who reported GIB but had insufficient medical records, died after reporting GIB, or refused permission for record review (n = 442). Our results remained similar. The risk of overall GIB was 1.34 (95% CI, 0.89–2.02) in men who consumed >30 g/day, and 1.55 (95% CI, 1.09–2.21) in men who consumed 15–29 g/day compared to non-drinkers.

### GIB and Smoking

Past smoking was not significantly associated with risk of overall GIB (multivariable RR 1.11; 95% CI 0.86–1.42) (Table 4). Likewise, current smokers were not at increased risk of GIB (multivariable RR 0.90; 95% CI, 0.45–1.79). There was no significant association between smoking status and risk of upper or lower GIB. We did not find a significant association between pack-years of smoking and risk of GIB (multivariable RR 1.06; 95% CI, 0.66–1.70, *P* trend = 0.46 when comparing men with at least 45 pack-years of exposure to never smokers), although there were few men who had smoked more than 45 pack-years (Table 5). When the two highest categories of smoking exposure were combined, we again found no significant association between smoking and risk of GIB.

**Table 4:** Smoking Status and Risk of Major GI Bleeding.

	Smoking Status		
	Never	Past	Current
Person-years	373001	369571	51146
**All GI bleeding**^**a**^			
No. of cases	114	152	9
Age-adjusted HR (95% CI)^b^	1.0	1.19 (0.93, 1.52)	0.91 (0.46, 1.80)
Multivariable HR (95% CI)^c^	1.0	1.11 (0.86, 1.42)	0.90 (0.45, 1.79)
Multivariable 2 HR (95% CI)^d^	1.0	1.07 (0.83, 1.37)	0.90 (0.65, 1.50)
**Upper GI bleeding**^**e**^			
No. of cases	59	65	4
Age-adjusted HR (95% CI)^b^	1.0	0.99 (0.69, 1.40)	0.75 (0.27, 2.08)
Multivariable HR (95% CI)^c^	1.0	0.90 (0.63, 1.30)	0.69 (0.25, 1.93)
Multivariable 2 HR (95% CI)^d^	1.0	0.87 (0.60, 1.25)	0.67 (0.41, 1.49)
**Lower GI bleeding**^**e**^			
No. of cases	43	67	4
Age-adjusted HR (95% CI)^b^	1.0	1.37 (0.93, 2.01)	1.13 (0.40, 3.16)
Multivariable HR (95% CI)^c^	1.0	1.33 (0.90, 1.97)	1.21 (0.43, 3.41)
Multivariable 2 HR (95% CI)^d^	1.0	1.29 (0.87, 1.90)	1.23 (0.44, 3.48)

NOTE: The total number of cases in the smoking status analysis differs from the alcohol analysis (n = 275 vs n = 305) due to the number of men with missing values for smoking and alcohol at baseline and the use of simple vs. cumulative updating, respectively.

^a^ Includes 24 cases of bleeding of unknown etiology and 9 cases of small bowel bleeding

^b^ Adjusted for age in years and study period in 2-year intervals and using simple updating to examine smoking status.

^c^ Adjusted for alcohol (none, 1–4 g/day, 5–14 g/day, 15–29 g/day, 30+ g/day), body mass index (<21, 25–29, 30–31, ≥32 kg/m^2^), physical activity (quintiles), regular use of aspirin (yes/no), regular use of NSAIDs (yes/no); using simple updating to examine smoking status.

^d^ Adjusted for multivariate model 1 plus medication use (proton pump inhibitors, H2 receptor antagonists, selective serotonin reuptake inhibitors, warfarin and/or clopidogrel) and comorbid disease (myocardial infarction, stroke or transient ischemic attack, rheumatoid arthritis, degenerative joint disease, peripheral vascular disease, chronic obstructive pulmonary disease, chronic kidney disease).

^e^ Upper GI bleeding was defined as bleeding originating from the esophagus, stomach, or duodenum; lower GI bleeding was defined as bleeding arising from the colon or rectum.

**Table 5:** Pack-years of Smoking and Risk of Major GI Bleeding.

	Pack-years of Smoking					
	0	< 10	10–24	25–44	≥45	P value for linear trend^a^
Person-years	421538	90534	165941	112604	58570	
**All GI bleeding**^**b**^						
No. of cases	123	28	59	53	21	
Age-adjusted HR (95% CI)^c^	1.0	1.06 (0.70, 1.60)	1.15 (0.85, 1.58)	1.52 (1.10, 2.10)	1.20 (0.75, 1.91)	0.33
Multivariable 1 HR (95% CI)^d^	1.0	1.02 (0.68, 1.55)	1.09 (0.79, 1.49)	1.38 (0.99, 1.92)	1.06 (0.66, 1.70)	0.46
Multivariable 2 HR (95% CI)^e^	1.0	1.02 (0.67, 1.54)	1.06 (0.77, 1.45)	1.30 (0.93, 1.81)	1.03 (0.64, 1.65)	0.34
**Upper GI bleeding**^**f**^						
No. of cases	62	8	26	26	11	
Age-adjusted HR (95% CI)^c^	1.0	0.60 (0.29, 1.25)	1.01 (0.64, 1.61)	1.48 (0.94, 2.35)	1.24 (0.65, 2.37)	0.24
Multivariable 1 HR (95% CI)^d^	1.0	0.56 (0.27, 1.18)	0.95 (0.60, 1.52)	1.34 (0.83, 2.14)	1.05 (0.54, 2.02)	0.33
Multivariable 2 HR (95% CI)^e^	1.0	0.56 (0.27, 1.18)	0.94 (0.59, 1.50)	1.27 (0.79, 2.04)	0.99 (0.51, 1.91)	0.42
**Lower GI bleeding**^**f**^						
No. of cases	46	18	26	18	7	
Age-adjusted HR (95% CI)^c^	1.0	1.83 (1.06, 3.16)	1.35 (0.83,2.18)	1.36 (0.79, 2.35)	1.05 (0.47, 2.33)	0.71
Multivariable 1 HR (95% CI)^d^	1.0	1.85 (1.07, 3.20)	1.31 (0.80, 2.13)	1.27 (0.73, 2.21)	0.98 (0.44, 2.21)	0.56
Multivariable 2 HR (95% CI)^e^	1.0	1.83 (1.05, 3.17)	1.27 (0.78, 2.07)	1.20 (0.69, 2.09)	0.98 (0.44, 2.21)	0.92

NOTE: The total number of cases in the pack years of smoking analysis differs from the alcohol analysis (n = 284 vs n = 305) due to the number of men with missing values for smoking and alcohol at baseline.

^a^ *P* value for trend calculated using the median value in each category of alcohol consumption as a continuous variable in the regression models

^b^ Includes 27 cases of bleeding of unknown etiology and 9 cases of small bowel bleeding

^c^ Adjusted for age in years and study period in 2-year intervals and using simple updating to examine smoking status.

^d^ Adjusted for alcohol (none, 1–4 g/day, 5–14 g/day, 15–29 g/day, 30+ g/day), body mass index (<21, 25–29, 30–31, ≥32 kg/m^2^), physical activity (quintiles), regular use of aspirin (yes/no), regular use of NSAIDs (yes/no); using simple updating to examine smoking status.

^e^ Adjusted for multivariate model 1 plus medication use (proton pump inhibitors, H2 receptor antagonists, selective serotonin reuptake inhibitors, warfarin and/or clopidogrel) and comorbid disease (myocardial infarction, stroke or transient ischemic attack, rheumatoid arthritis, degenerative joint disease, peripheral vascular disease, chronic obstructive pulmonary disease, chronic kidney disease).

^f^ Upper GI bleeding was defined as bleeding originating from the esophagus, stomach, or duodenum; lower GI bleeding was defined as bleeding arising from the colon or rectum

## Discussion

In this large, prospective study of men, we observed that alcohol consumption, even in modest amounts, was associated with increased risk of major GIB. This finding was strongest for liquor consumption and upper GIB secondary to peptic ulcer disease. Men who consumed alcohol without meals were at particularly high risk of upper GIB. Alcohol consumption potentiated the risk of GIB associated with regular use of NSAIDs and/or aspirin. In contrast, smoking did not appear to be associated with increased risk of any type of major GIB.

Several studies have examined the association between alcohol consumption and major GIB. [4–7,9–11] However, most of these studies utilized case-control designs and were not designed specifically to assess the association of alcohol consumption with GIB. [6,7,9,11] In addition, most of these studies evaluated the association of alcohol consumption only in relation to specific etiologies of GIB (e.g. peptic ulcer, diverticular) rather than overall GIB, which is an endpoint of substantial interest to clinicians.[4,5,9,11,13] Only a few studies have reported the association of alcohol consumption on upper GIB.[4,16,30–33] Some, [4,16,33] but not all have demonstrated a significant association.[30–32] Most of these studies are limited by small sample size, recall bias, and a lack of a definition of “alcohol exposure.” [30–33] Furthermore, studies that defined alcohol exposure largely examined heavy alcohol use.[16,34] In contrast, our study, suggests that even moderate alcohol consumption increases the risk of major GIB. In addition, our findings indicate that the association between alcohol and major GIB is specific to consumption of liquor and to those who drink alcohol without meals.

A number of studies have also examined the association between alcohol consumption and specific etiologies of bleeding, particularly peptic ulcer disease and diverticular bleeding. [5,9–11,13] In a prospective cohort study, Andersen *et al* found a four-fold increased risk of bleeding peptic ulcer in individuals consuming > 42 drinks/week compared with those consuming less than one drink/week.[5] We also found a significant association between alcohol consumption and bleeding from peptic ulcers, although again we noted that even moderate alcohol consumption can increase risk. Consistent with our findings, a pair of case-control studies did not find any significant association between alcohol consumption and risk of diverticular bleeding. [9,13]

Although the risk of GIB associated with regular use of NSAID and aspirin use is well established, it is unclear if concurrent alcohol consumption modifies this risk.[21,35,36] In a study of 20 healthy male volunteers, mean fecal blood loss measured via red blood cell radiolabeling was significantly higher when aspirin was ingested with alcohol than when aspirin was taken alone.[37] A case-control study, found that the risk of upper GIB associated with aspirin increased with heavy alcohol consumption (at least 20 drinks/week) although a multivariate risk estimate could not be calculated due to the small number of cases in this category, and an interaction was not seen with lesser amounts of alcohol consumption.[38] In the present analysis, we found that the risk of GIB associated with NSAID/aspirin use increases with greater alcohol consumption.

In addition, we found that men who consumed alcohol without meals were at greater risk of GIB, especially UGIB, compared to men who consumed the same amount of alcohol with meals. This finding may explain why liquor consumption is specifically associated with elevated risk of upper GIB compared with wine or beer, even after accounting for the amount of alcohol within each beverage type. Liquor, compared with beer or wine, may be more likely to be consumed separately from meals.

Our findings are biologically plausible. Alcohol has been shown to cause exfoliation of gastric epithelium, edema of the lamina propria, necrosis of deeper tissue layers, and hemorrhagic erosions associated with microvascular damage.[39] Mucosal injury due to alcohol may be due to overproduction of oxygen free radicals, decreased prostaglandin synthesis, as well as release of mucosal leukotrienes and constriction of sub-mucosal venules.[40–45] Alcohol-related acute gastric injury in dogs is potentiated by nonsteroidal anti-inflammatory drugs.[46] Finally, certain foods and food components particularly those high in anti-oxidant properties also have been shown to protect against ethanol-induced injury.[47–49] In addition, the ingestion of food with alcohol slows the rate of alcohol absorption and decreases blood alcohol concentrations.[50] Therefore, food may modify the risk of bleeding associated with alcohol through systemic mechanisms. For example, alcohol has a number of deleterious effects on hemostasis, and bleeding time and blood loss are increased following acute ethanol administration in animal models.[51,52]

We did not find any association between smoking and GIB. Our results are in agreement with some, [9,11] but not all case-control studies on this topic. [6,7,10,13] However, these studies are limited by evaluation of only a few specific GIB etiologies. [9,13] In a prospective cohort study, Kaplan *et al* reported that current smokers had a higher risk of hospitalization for upper GIB (but not lower GIB) than nonsmokers, and that this relationship was characterized by an increasing dose-response pattern.[8] However, their population was older than 65 years and may not be generalizable to a broader population. In a separate prospective cohort study, Andersen et al, observed that smoking was not associated with bleeding from peptic ulcers.[5] *H*. *pylori* may modify the effect of smoking on peptic ulcer disease. Therefore, the conflicting literature on smoking and risk of GIB could reflect in part differences in *H*. *pylori* prevalence.

Our study has several important strengths. First, we prospectively collected data on alcohol consumption and smoking prior to diagnosis of GIB which minimized the potential for recall bias. Second, we collected detailed data on alcohol consumption and smoking for more than 25 years of follow-up enabling a more stable estimate of long-term exposure than in prior studies. Third, we had detailed data on a broad range of other factors associated with GIB, including concomitant medication use (aspirin, NSAIDs, proton pump inhibitors, H2 receptor antagonists, selective serotonin reuptake inhibitors, clopidogrel and warfarin) as well as), lifestyle factors and comorbid illness. Fourth, we reviewed medical records to confirm reported cases of GIB, minimizing the potential for misclassification of our outcomes. We also acknowledge several limitations of our study. Both alcohol consumption and smoking were self-reported. However, self-report of alcohol consumption has been previously validated in this cohort. [23] We did not have information on the *H*. *pylori* status of all study subjects. However, evidence suggests that the consumption of alcohol does not affect *H*. *pylori* infection.[53,54] Furthermore, *H*. *pylori* infection is likely to be treated when identified in clinical practice and we excluded individuals with a history of peptic ulcer disease, including those presumably with ulcers that were H pylori related. Our study cohort is comprised of male health professionals that may limit the generalizability of our study. In addition, HPFS participants tend to consume less alcohol than the general population. Therefore, we had limited power to assess heavy alcohol consumption. Similarly, current smoking was relatively infrequent in our cohort so our power was limited. We had more power to examine smoking in pack years, but most of this exposure was remote from the GIB events. It is also likely that some cases of GIB were missed due to underreporting. This is particularly true during the early years of the cohort as GIB was not ascertained until 2006. However, cases missed in early study years are unlikely to be biased with respect to alcohol or smoking exposure. Finally, although we adjusted for a broad range of risk factors for GIB we are unable to exclude the possibility that residual confounding may have biased our result.

## Conclusions

In conclusion, in this large prospective study, consumption of >15 g/day of alcohol (approximately 1 drink or more) was associated with an increased risk of upper GIB, particularly secondary to peptic ulcer. The risk was elevated primarily among men who consumed alcohol separately from meals. Alcohol appeared to potentiate the known effect of NSAIDS and aspirin on risk of GIB. In contrast, smoking was not associated with risk of GIB. Our results suggest that beyond the known association of alcohol with cirrhosis, alcohol may also be a modifiable risk factor for non-portal hypertensive causes of GIB, including NSAID-related bleeding and peptic ulcer disease. Clinically, this provides evidence for moderation in the consumption of alcohol.

## Supporting Information

S1 Questionnaire 2006 Biennial Questionnaire. (PDF)

S1 Table Multivariable RRs and 95% CIs of GI bleeding according to alcohol intake and use of nonsteroidal anti-inflammatory drugs. (DOCX)

S2 Table Alcohol Consumption and Risk of Major GI Bleeding According to Bleeding Source. (DOCX)
