# Supplementary material for: A Prospective Study of Alcohol Consumption and Smoking and the Risk of Major Gastrointestinal Bleeding in Men
Source: PLoS One. 2016 Nov 8;11(11):e0165278. doi: 10.1371/journal.pone.0165278 (PMC5100927; doi:10.1371/journal.pone.0165278)
Supplement: S1 Questionnaire — (PDF) [file pone.0165278.s001.pdf]

HARVARD SCHOOL OF PUBLIC HEALTH

Health Professionals Follow-up Study

Advisory Board

Chester Douglass, D.D.S.
Harvard School of Dental Medicine

William A. Gouveia, M.S.
American Society of Health-System Pharmacists

Melvyn Grovit, D.P.M.
American Podiatric Medical Association

Janet Donlin, D.V.M.
American Veterinary Medical Association

J. Peter Tilley, D.O.
Philadelphia College of Osteopathic Medicine

John Whitener, O.D.
American Optometric Association

Susan C. Winckler, R.Ph. (Pharm)
American Pharmaceutical Association

Research Staff

Walter C. Willett, M.D.
Principal Investigator

Jill Arnold
Alberto Ascherio, M.D.
Luba Bondarenko
Gary Curhan, M.D.
Immaculata De Vivo, Ph.D.
Stacey DeCaro
Rachael DePasquale
Lauren Dougherty
Mary Franz, R.D.
Betsy Frost-Hawes
Charles Fuchs, M.D.
Edward Giovannucci, M.D.
Frank B. Hu, M.D.
Cheryl Jones
Christine Jones
Mira Kaufman
Ichiro Kawachi, M.D.
Elizabeth Lenart, Ph.D.
Jetdell Lo-Pinto
Dominique Michaud, Sc.D.
Elizabeth Platz, Sc.D.
Eric Rimm, Sc.D.
Siobhan Saint-Surin
Laura Sampson, R.D.
Robert Sheahan
Stephanie Smith-Warner, Ph.D.
Donna Spiegelman, Sc.D.
Meir Stampfer, M.D.
Adam Summerfield
Annya Tisher
Paula Tocco, R.D.
Olga Veysman
Carol Willey, R.D.
Al Wing
Mitzi Wolff
Kana Wu, M.D., Ph.D.

Dear Colleagues,

This questionnaire marks the 20-year point in the Health Professionals Follow-Up Study, which began in 1986. During this time we have learned much about ways that diet and lifestyle factors can help reduce our risks of heart disease, stroke, and cancer, and promote healthy aging. This has only been possible because of your remarkable dedication, and that of other participants, to this research. The response to our 2004 questionnaire remained well above 90%, ensuring the validity of information from this investigation.

During the past several years, new insights on risk factors for prostate and colon cancer have emerged from this study. Among men 65 years or older, higher consumption of lycopene(1,2) (the red pigment in tomatoes), fish(3) as well as vigorous physical activity(4) were each associated with a lower risk of prostate cancer. Conversely, high doses of zinc supplements (100 mg per day or more) were related to increased risk of prostate cancer.(5) Although some earlier studies had suggested higher risks of colon cancer with greater coffee consumption, we found no association.(6) Aspirin use was associated with lower risk of colon adenomas, precursors for colon cancer, particularly among men who have a slow-acting form of a gene responsible for metabolizing aspirin.(7) This finding needs replication, but suggests that it may be possible to identify individuals who would most benefit by the use of aspirin for cancer prevention. Further details on our findings will be included in our newsletter next year and are available on our website (www.hsph.harvard.edu/hpfs).

The attached 2006 questionnaire continues the critical follow-up of this study. Most importantly, we request information about the diagnosis of specific diseases since January 1, 2004. As always, all information provided on this questionnaire is strictly confidential and is used only for statistical purposes.

Again, I thank you for your participation in this research, which continues to provide new information on ways to reduce major illness in men.

Sincerely,

Walter Willett

Walter Willett, MD
Principal Investigator

1. **JNCI,** 2002, Vol. 94, p. 391 (A prospective study of tomato products, lycopene, and prostate cancer risk).
2. **Cancer Epidemiol Biomarkers Prev,** 2004, Vol. 13, p. 260 (Plasma and dietary carotenoids, and the risk of prostate cancer: A nested case-control study).
3. **Cancer Epidemiol Biomarkers Prev,** 2003, Vol. 12, p. 64 (A prospective study of intake of fish and marine fatty acids and prostate cancer).
4. **Arch Intern Med, 2005,** Vol. 165, p. 1005 (A prospective study of physical activity and incident and fatal prostate cancer).
5. **JNCI,** 2003, Vol. 95, p. 1004 (Zinc supplement use and risk of prostate cancer).
6. **JNCI,** 2005, Vol. 97, p. 282 (Coffee, tea, and caffeine consumption and incidence of colon and rectal cancer).
7. **JNCI,** 2005, Vol. 97, p. 457 (Genetic variants in the UGT1A6 enzyme, aspirin use, and the risk of colorectal adenoma).

Please reply to: HSPH 677 Huntington Avenue, Boston MA 02115-5804 • (617) 998-1067

3/8" spine perf

USE NO. 2 PENCIL ONLY

PLEASE USE AN ORDINARY NO. 2 PENCIL TO ANSWER ALL QUESTIONS.

Fill in the appropriate response circles completely, or write the requested information in the boxes provided. The form is designed to be read by optical-scanning equipment, so it is important that you make NO STRAY MARKS and keep any write-in responses within the spaces provided. Should you need to change a response, erase the incorrect mark completely. If you have comments, please write them on a separate piece of paper.

EXAMPLE 1: 23. **Do you currently take multi-vitamins?**

- ○ No
- ● Yes

Please fill circle completely, do not mark this way: ✓ ✗ ◒

EXAMPLE 2: b) What specific brand (or equivalency) do you usually take?

CVS Daily Multivitamin with Minerals

e.g., AARP Alphabet II Formula 643 Multivitamins and Minerals

Keep handwriting within borders of the response box.

Federal research regulations require us to include the following information:
There are no direct benefits to you from participating in this study.
The risk of breach of confidentiality associated with participation in this study is very small.
Your choice to participate in this study is completely voluntary and you may decline or withdraw at any time without penalty.
You may skip any question you do not wish to answer.
You will not receive monetary compensation for participating.
If you have any questions regarding your rights as a research participant, you are encouraged to call a representative of the Human Subjects Committee at the Harvard School of Public Health (866-606-0573).
If you have any questions regarding your status in our study or a question pertaining to the questionnaire, please call the study Project Coordinator, Betsy Frost-Hawes, at 617-384-8657.

Thank you for completing the 2006 Health Professionals Follow-up Study questionnaire.

Please tear off the cover letter (to preserve confidentiality) and return the questionnaire in the enclosed prepaid envelope.

3/8" spine perf

© Copyright 2006 by President and Fellows of Harvard College. All Rights Reserved Worldwide.

1. What is your current weight (pounds)?

2. Current Marital Status: ◯ Married ◯ Divorced/Separated ◯ Widowed ◯ Never married

3. Living Arrangement: ◯ Alone ◯ With wife ◯ With other family ◯ Assisted living ◯ Nursing home ◯ Other

4. Work Status: ◯ Full-time ◯ Part-time ◯ Retired ◯ Disabled ◯ Unemployed

5. Do you currently smoke cigarettes? (exclude pipe or cigars)
◯ No ◯ Yes → Please mark your average number of cigarettes per day:
◯ 1–4 cigarettes ◯ 5–14 ◯ 15–24 ◯ 25–34 ◯ 35–44 ◯ 45 or more

6. In the past 2 years, have you had . . .

… a physical exam?	◯ No	◯ Yes, for symptoms	◯ Yes, for routine screening
… a rectal exam?	◯ No	◯ Yes, for symptoms	◯ Yes, for routine screening
… an eye exam?	◯ No	◯ Yes, for symptoms	◯ Yes, for routine screening
… blood glucose check?	◯ No	◯ Yes, for symptoms	◯ Yes, for routine screening
… screening for PSA?	◯ No	◯ Yes, for symptoms	◯ Yes, for routine screening

If "yes" for PSA screening, was your PSA elevated? ◯ No ◯ Unknown ◯ Yes
… a prostate biopsy or rectal ultrasound (for prostate exam)? ◯ No ◯ Unknown ◯ Yes
… upper endoscopy (esophagus/stomach)? ◯ No ◯ Yes
… a colonoscopy? ◯ No ◯ Yes
… a sigmoidoscopy? ◯ No ◯ Yes

6a. Initial reason(s) you had a colonoscopy/sigmoidoscopy?
◯ Visible blood ◯ Occult fecal blood ◯ Abdominal pain
◯ Family history of colon cancer ◯ Diarrhea/constipation ◯ Barium enema
◯ Virtual (CT) colonography ◯ Prior polyps ◯ Asymptomatic or routine screening

7. Have you ever had gastrointestinal bleeding that required hospitalization or a transfusion?
◯ No ◯ Yes ▶ a) Sites: ◯ Esophagus ◯ Stomach ◯ Duodenum ◯ Colon/Rectum ◯ Other ◯ Site(s) unknown
b) What year(s)? (Mark all that apply) ◯ Before 1993 ◯ '93–'95 ◯ '96–'97 ◯ '98–'99 ◯ 2000–'01 ◯ 2002–'03 ◯ 2004+

8. Do you have a hearing problem? ◯ No ◯ Mild ◯ Moderate ◯ Marked, no hearing aid ◯ Severe, use a hearing aid

9. In a typical week during the past year, on how many days did you consume an alcoholic beverage of any type?
◯ No days ◯ 1 day/week ◯ 2 days/week ◯ 3 days/week
◯ 4 days/week ◯ 5 days/week ◯ 6 days/week ◯ 7 days/week

10. In a typical month, what is the largest number of drinks of beer, wine and/or liquor you have in one day?
◯ None ◯ 1–2 drinks/day ◯ 3–5 ◯ 6–9 ◯ 10–14 ◯ 15 or more drinks/day

11. How many times per day do you eat? Include meals and snacks. (For snacks, count juice and non-diet soda, but exclude coffee and diet soda.)
◯ 1 or 2 times per day ◯ 3/day ◯ 4/day ◯ 5/day ◯ 6/day ◯ 7/day ◯ 8/day ◯ 9 or more times per day

12. What percent of your noon and evening meals are prepared at home? (Exclude commercially prepared meals.)
◯ Almost none ◯ 25% ◯ 50% ◯ 75% ◯ Almost all

13. How many teeth have you lost since January 1, 2004? ◯ None ◯ 1 ◯ 2 ◯ 3 ◯ 4 ◯ 5–9 ◯ 10+

14. Do you have difficulty with your balance? ◯ No ◯ Yes

15. Do you have difficulty climbing a flight of stairs or walking eight blocks due to a physical impairment? ◯ No ◯ Yes

16. How many flights of stairs (not steps) do you climb daily? (Do not include time spent on stair or exercise machines.)
◯ No flights ◯ 1–2 flights ◯ 3–4 flights ◯ 5–9 flights ◯ 10–14 flights ◯ 15 or more flights

17. During the past year, what was your average total time per week at each activity?

AVERAGE TOTAL TIME PER WEEK

Activity	NONE	1–4 Min.	5–19 Min.	20–39 Min.	40–80 Min.	1.5 Hrs.	2–3 Hrs.	4–6 Hrs.	7–10 Hrs.	11–20 Hrs.	21–30 Hrs.	31–40 Hrs.	40+ Hrs.
Sitting at work	◯	◯	◯	◯	◯	◯	◯	◯	◯	◯	◯	◯	◯
Sitting or driving (e.g., car, bus, or train)	◯	◯	◯	◯	◯	◯	◯	◯	◯	◯	◯	◯	◯
Sitting or lying watching TV or VCR	◯	◯	◯	◯	◯	◯	◯	◯	◯	◯	◯	◯	◯
Sitting at home reading	◯	◯	◯	◯	◯	◯	◯	◯	◯	◯	◯	◯	◯
Sitting at home working on a computer	◯	◯	◯	◯	◯	◯	◯	◯	◯	◯	◯	◯	◯
Other sitting at home (e.g., at desk or eating)	◯	◯	◯	◯	◯	◯	◯	◯	◯	◯	◯	◯	◯
Walking to work or for exercise (including golf)	◯	◯	◯	◯	◯	◯	◯	◯	◯	◯	◯	◯	◯
Jogging (slower than 10 minutes/mile)	◯	◯	◯	◯	◯	◯	◯	◯	◯	◯	◯	◯	◯
Running (10 minutes/mile or faster)	◯	◯	◯	◯	◯	◯	◯	◯	◯	◯	◯	◯	◯
Bicycling (including stationary machine)	◯	◯	◯	◯	◯	◯	◯	◯	◯	◯	◯	◯	◯
Lap swimming	◯	◯	◯	◯	◯	◯	◯	◯	◯	◯	◯	◯	◯
Tennis	◯	◯	◯	◯	◯	◯	◯	◯	◯	◯	◯	◯	◯
Squash or racquetball	◯	◯	◯	◯	◯	◯	◯	◯	◯	◯	◯	◯	◯
Calisthenics, rowing, stair or ski machine, etc.	◯	◯	◯	◯	◯	◯	◯	◯	◯	◯	◯	◯	◯
Weightlifting or weight machine	◯	◯	◯	◯	◯	◯	◯	◯	◯	◯	◯	◯	◯
Moderate outdoor work (e.g., yardwork, gardening)	◯	◯	◯	◯	◯	◯	◯	◯	◯	◯	◯	◯	◯
Heavy outdoor work (e.g., digging, chopping)	◯	◯	◯	◯	◯	◯	◯	◯	◯	◯	◯	◯	◯

Mark Reflex® forms by NCS Pearson EM-239729-2:654321 Printed in U.S.A.
3/8" spine perf

18. IS THIS YOUR CORRECT DATE OF BIRTH? (18)

- ○ Yes
- ○ No → IF NO, please indicate your date of birth. (a)

MONTH	DAY	YEAR

19. Since January 1, 2004, have you had any of the following clinician diagnosed conditions? (19)

Leave blank for NO, mark here for YES (a)

Condition	YES	YEAR OF DIAGNOSIS: Before 2004	2004	2005	2006	
High blood pressure	Ⓨ	○	○	○	○	1
Diabetes mellitus	Ⓨ	○	○	○	○	2
Elevated cholesterol	Ⓨ	○	○	○	○	3
Elevated triglycerides	Ⓨ	○	○	○	○	4
Coronary bypass, angioplasty or stent	Ⓨ	○	○	○	○	5
Myocardial infarction (heart attack)	Ⓨ	○	○	○	○	6
Hospitalized for this MI? ○ No ○ Yes						a
Angina pectoris	Ⓨ	○	○	○	○	7
Confirmed by angiogram? ○ No ○ Yes						a
Congestive heart failure	Ⓨ	○	○	○	○	8
Deep vein thrombosis	Ⓨ	○	○	○	○	9
TIA (Transient Ischemic Attack)	Ⓨ	○	○	○	○	10
Stroke (CVA)	Ⓨ	○	○	○	○	11
Carotid artery surgery	Ⓨ	○	○	○	○	12
Intermittent claudication	Ⓨ	○	○	○	○	13
Surgery or angioplasty for arterial disease of the leg	Ⓨ	○	○	○	○	14
Pulmonary embolus	Ⓨ	○	○	○	○	15
Aortic aneurysm	Ⓨ	○	○	○	○	16
Gout	Ⓨ	○	○	○	○	17
Rheumatoid arthritis	Ⓨ	○	○	○	○	18
Other arthritis (e.g., osteoarthritis)	Ⓨ	○	○	○	○	19
Chronic renal failure	Ⓨ	○	○	○	○	20
Diverticulitis or Diverticulosis	Ⓨ	○	○	○	○	21
Prostatic enlargement, surgically treated (e.g., TURP)	Ⓨ	○	○	○	○	22
Prostate cancer	Ⓨ	○	○	○	○	23
Colon or rectal polyp	Ⓨ	○	○	○	○	24
Cancer of colon or rectum	Ⓨ	○	○	○	○	25
Solar or actinic keratosis	Ⓨ	○	○	○	○	26
Basal cell skin cancer	Ⓨ	○	○	○	○	27
Squamous cell skin cancer	Ⓨ	○	○	○	○	28
Melanoma	Ⓨ	○	○	○	○	29
Lymphoma or Leukemia	Ⓨ	○	○	○	○	30
Other cancer	Ⓨ	○	○	○	○	31
Please specify site and year: →						a
Glaucoma	Ⓨ	○	○	○	○	32
Cataract (1st Diagnosis)	Ⓨ	○	○	○	○	33
Cataract extraction	Ⓨ	○	○	○	○	34
Macular degeneration	Ⓨ	○	○	○	○	35
Osteoporosis	Ⓨ	○	○	○	○	36
Hip replacement	Ⓨ	○	○	○	○	37
Periodontal disease with bone loss	Ⓨ	○	○	○	○	38
Leukoplakia/oral precancer	Ⓨ	○	○	○	○	39
Gall bladder removal	Ⓨ	○	○	○	○	40
Kidney stones	Ⓨ	○	○	○	○	41
Parkinson's disease	Ⓨ	○	○	○	○	42
ALS (Amyotrophic Lateral Sclerosis)	Ⓨ	○	○	○	○	43
Gastric or duodenal ulcer	Ⓨ	○	○	○	○	44
Barrett's esophagus	Ⓨ	○	○	○	○	45
Ulcerative colitis/Crohn's disease	Ⓨ	○	○	○	○	46

19. *(continued)* (19)

Leave blank for NO, mark here for YES (a)

Condition	YES	Before 2004	2004	2005	2006	
Shingles	Ⓨ	○	○	○	○	47
Seizure (1 or more)/epilepsy	Ⓨ	○	○	○	○	48
Alcohol dependence problem	Ⓨ	○	○	○	○	49
Pneumonia (X-ray confirmed)	Ⓨ	○	○	○	○	50
Asthma	Ⓨ	○	○	○	○	51
Pernicious Anemia/B12 deficiency	Ⓨ	○	○	○	○	52
Emphysema or chronic bronchitis (COPD)	Ⓨ	○	○	○	○	53
Other major illness or surgery since January 2004	Ⓨ	○	○	○	○	54

Please specify: (0 1 2 3 4 5 6 7 8 9) 55 / w

20. Have you ever been diagnosed as having atrial fibrillation (more than 1 hour)? (20)

○ No ○ Yes (w)

If Yes: ○ Before 1986 ○ 1986–94 ○ 1994–2004 ○ 2005 ○ 2006

21. Since January 1, 2004, have you had any of these fractures? (21)

○ None ○ Hip *(exclude pelvis)* ○ Wrist *(Colles or distal forearm)*

If hip or wrist, please specify date and circumstances. If a fall, include site, surface and height of fall.

Month: ________
Year: ________

22. Current Medication (mark if used regularly) (22)

- ○ Acetaminophen (e.g., Tylenol)
  - **Days/week:** ○ 1 ○ 2–3 ○ 4–5 ○ 6+ days
  - **Tablets/wk:** ○ 1–2 ○ 3–5 ○ 6–14 ○ 15+ tablets
- ○ Aspirin or aspirin-containing products (e.g., Alka-Seltzer with aspirin)
  - **Days/week:** ○ 1 ○ 2–3 ○ 4–5 ○ 6+ days
  - **Tablets/wk:** ○ 1–2 ○ 3–5 ○ 6–14 ○ 15+ tablets
  - **Usual dose/tab:** ○ 50–99 mg ○ 100–249 ○ 250–349 ○ 350+
- ○ Ibuprofen (e.g., Advil, Motrin, Nuprin)
  - **Days/week:** ○ 1 ○ 2–3 ○ 4–5 ○ 6+ days
  - **Tablets/wk:** ○ 1–2 ○ 3–5 ○ 6–14 ○ 15+ tablets
- ○ Celebrex (COX-2 inhibitors)
  - **Days/week:** ○ 1 ○ 2–3 ○ 4–5 ○ 6+ days
- ○ Other anti-inflammatory analgesics, 2+ times/week (e.g., Aleve, Naprosyn, Anaprox, Relafen, Ketoprofen)
- ○ Steroid taken orally (e.g., Prednisone, Medrol)
- ○ "Statin" cholesterol-lowering drug:
  - ○ Mevacor (lovastatin) ○ Zocor (simvastatin) ○ Crestor
  - ○ Pravachol (pravastatin) ○ Lipitor (atorvastin) ○ Other
- ○ Other cholesterol-lowering drug [e.g., niacin, Lopid (gemfibrozil), Tricor (fenofibrate), Questran (cholestyramine), Colestid, Zetia]
- ○ H2 blocker (e.g., Pepcid, Tagamet, Zantac, Axid)
- ○ Prilosec, Nexium, Prevacid, Protonix, Aciphex
- ○ Finasteride (e.g., Proscar, Propecia, Avodart)
- ○ Alpha-blocker for BPH [e.g., Hytrin (terazosin), Flomax, Cardura]
- ○ Beta-blocker (e.g., Inderal, Metoprolol, Atenolol, Carvedilol)
- ○ ACE inhibitor or ARB (e.g., Prinivil, Vasotec, Diovan Avapro)
- ○ Furosemide-like diuretic (e.g., Lasix, Bumex)
- ○ Thiazide diuretic (e.g., HCTZ, Maxzide, Dyazide)
- ○ Calcium blocker (e.g., Calan, Procardia, Cardizem, Norvasc)
- ○ Other antihypertensive (e.g., Clonidine, Aldactone)
- ○ Prozac, Zoloft, Paxil, Celexa, Effexor
- ○ Tricyclic antidepressant (e.g., Elavil, Sinequan)
- ○ Other antidepressant (e.g., Trazodone, Nardil)
- ○ Tranquilizer (e.g., Valium, Xanax, Klonopin)
- ○ Coumadin (e.g., Warfarin)
- ○ Digoxin (e.g., Lanoxin)
- ○ Other regular medication (no need to specify)

3/8" spine perf

23. Do you currently take multi-vitamins? (Please report other individual vitamins in the next section.)

○ No ○ Yes → a) How many do you take per week? ○ 2 or less ○ 3–5 ○ 6–9 ○ 10 or more

b) What specific brand (or equivalency) do you usually take?

○ Centrum Silver ○ Centrum ○ Other →
○ Theragran M ○ One-A-Day Essential

e.g., AARP Alphabet II Formula 643 Multivitamins and Minerals

Not counting multi-vitamins, do you take any of the following preparations?

Preparation									
a) Vitamin A	○ No	○ Yes, seasonal only ○ Yes, most months	If Yes,	Dose per day:	○ Less than 8,000 IU	○ 8,000 to 12,000 IU	○ 13,000 to 22,000 IU	○ 23,000 IU or more	○ Don't know
b) Potassium (over the counter)	○ No	○ Yes	If Yes,	Dose per day:	○ Less than 2.5 mEq (100 mg)	○ 3 to 10 mEq	○ 11 to 20 mEq	○ 21 mEq or more	○ Don't know
c) Vitamin C	○ No	○ Yes, seasonal only ○ Yes, most months	If Yes,	Dose per day:	○ Less than 400 mg	○ 400 to 700 mg	○ 750 to 1250 mg	○ 1300 mg or more	○ Don't know
d) Vitamin B_6	○ No	○ Yes	If Yes,	Dose per day:	○ Less than 25 mg	○ 26 to 50 mg	○ 51 to 100 mg	○ 101 mg or more	○ Don't know
e) Vitamin E	○ No	○ Yes	If Yes,	Dose per day:	○ Less than 100 IU	○ 100 to 250 IU	○ 300 to 500 IU	○ 600 IU or more	○ Don't know
			Type:	○ Natural	○ Regular (dl)	○ Unknown			
f) Calcium (Include Calcium in Tums, etc.) (1 Tum = 200 mg elemental calcium)	○ No	○ Yes	If Yes,	Dose per day (elemental calcium):	○ Less than 500 mg	○ 501 to 1000 mg	○ 1001 to 1500 mg	○ 1501 mg or more	○ Don't know
g) Selenium	○ No	○ Yes	If Yes,	Dose per day:	○ Less than 80 mcg	○ 80 to 130 mcg	○ 140 to 250 mcg	○ 260 mcg or more	○ Don't know
h) Coenzyme Q10	○ No	○ Yes	If Yes,	Dose per day:	○ Less than 30 mg	○ 30 to 100 mg	○ 101 to 200 mg	○ 201 mg or more	○ Don't know
i) Zinc	○ No	○ Yes	If Yes,	Dose per day:	○ Less than 25 mg	○ 25 to 74 mg	○ 75 to 100 mg	○ 101 mg or more	○ Don't know

Are there other supplements that you take on a regular basis?

○ Metamucil/Citrucel ○ Beta-carotene ○ Chromium ○ Folic Acid ○ DHEA ○ Vitamin D
○ Cod Liver Oil ○ Magnesium ○ Lecithin ○ B-Complex ○ Iron ○ Other (Please specify)
○ Vitamin B_{12} ○ Melatonin ○ Saw Palmetto ○ Ginkgo Biloba
○ Niacin ○ Fish oil ○ Glucosamine ○ Garlic Supplements
○ Ginseng ○ St. John's Wort ○ Chondroitin ○ Lycopene

24. How many teaspoons of sugar do you add to your beverages or food each day? → ______ tsp.

25. What brand and type of cold breakfast cereal do you usually eat? →

Specify cereal brand & type (e.g., Kellogg's Raisin Bran)

○ Don't eat cold breakfast cereal.

26. What form of margarine do you usually use? →

○ None **Form?** ○ Stick ○ Tub ○ Spray ○ Squeeze *(liquid)*
Type? ○ Reg ○ Light ○ Nonfat

What specific brand & type of margarine (e.g., Shedd's Spread Country Crock Light Tub)

27. For each food listed, fill in the circle indicating how often on average you have used the amount specified during the past year.

Ch rb cf sw gn t k w

DAIRY FOODS		Never, or less than once per month	1–3 per month	1 per week	2–4 per week	5–6 per week	1 per day	2–3 per day	4–5 per day	6+ per day
Milk (8 oz. glass)	Skim milk	○	○	W	○	○	D	○	○	○
	1 or 2 % milk	○	○	W	○	○	D	○	○	○
	Whole milk	○	○	W	○	○	D	○	○	○
	Soy milk	○	○	W	○	○	D	○	○	○
Cream, e.g., coffee, whipped or sour cream (1 Tbs)		○	○	W	○	○	D	○	○	○
Non-dairy coffee whitener (1 Tbs)		○	○	W	○	○	D	○	○	○
Frozen yogurt, sherbet or low-fat ice cream (1 cup)		○	○	W	○	○	D	○	○	○
Regular ice cream (1 cup)		○	○	W	○	○	D	○	○	○
Yogurt (1 cup)	Low-carb, artificially sweetened or plain	○	○	W	○	○	D	○	○	○
	Sweetened–with fruit or other flavoring	○	○	W	○	○	D	○	○	○
Margarine (pat), added to food or bread; exclude use in cooking		○	○	W	○	○	D	○	○	○
Butter (pat), added to food or bread; exclude use in cooking		○	○	W	○	○	D	○	○	○
Cottage or ricotta cheese (1/2 cup)		○	○	W	○	○	D	○	○	○
Cream cheese (1 oz.)		○	○	W	○	○	D	○	○	○
Other cheese, e.g., American, cheddar, etc., plain or as part of a dish (1 slice or 1 oz. serving)		○	○	W	○	○	D	○	○	○

What type of cheese do you usually eat? ○ Regular ○ Low fat or Lite ○ Nonfat ○ None

3/8" spine perf

27. ***(continued)*** **For each food listed, fill in the circle indicating how often on average you have used the amount specified during the past year.**

Please try to average your seasonal use of foods over the entire year. For example, if a food such as cantaloupe is eaten 4 times a week during the approximate 3 months that it is in season, then the average use would be once per week.

FRUITS	Never, or less than once per month	1–3 per month	1 per week	2–4 per week	5–6 per week	1 per day	2–3 per day	4–5 per day	6+ per day
Raisins (1 oz. or small pack) or grapes (1/2 cup)									
Prunes or dried plums (6 prunes or 1⁄4 cup)									
Prune juice (small glass)									
Bananas (1)									
Cantaloupe (1/4 melon)									
Avocado (1/2 fruit or 1/2 cup)									
Fresh apples or pears (1)									
Apple juice or cider (small glass)									
Oranges (1)									
Orange juice (small glass): Calcium fortified									
Orange juice (small glass): Regular (not calcium fortified)									
Grapefruit (1/2) or grapefruit juice (small glass)									
Other fruit juices (small glass)									
Strawberries, fresh, frozen or canned (1/2 cup)									
Blueberries, fresh, frozen or canned (1/2 cup)									
Peaches or plums (1 fresh or 1/2 cup canned)									
Apricots, 1 fresh, 1/2 cup canned or 5 dried									

VEGETABLES	Never, or less than once per month	1–3 per month	1 per week	2–4 per week	5–6 per week	1 per day	2–3 per day	4–5 per day	6+ per day
Tomatoes (2 slices)									
Tomato or V-8 juice (small glass)									
Tomato sauce (1/2 cup) e.g., spaghetti sauce									
Salsa, picante or taco sauce (1/4 cup)									
String beans (1/2 cup)									
Beans or lentils, baked or dried (1/2 cup)									
Tofu, soy burger, soybeans, miso or other soy protein									
Peas or lima beans (1/2 cup fresh), frozen, canned)									
Broccoli (1/2 cup)									
Cauliflower (1/2 cup)									
Cabbage or coleslaw (1/2 cup)									
Brussels sprouts (1/2 cup)									
Carrots, raw (1/2 carrot or 2–4 sticks)									
Carrots, cooked (1/2 cup) or carrot juice (2–3 oz.)									
Corn (1 ear or 1/2 cup frozen or canned)									
Mixed or stir-fry vegetables (1/2 cup), veg. soup (1 cup)									
Yams or sweet potatoes (1/2 cup)									
Dark orange (winter) squash (1/2 cup)									
Eggplant, zucchini or other summer squash (1/2 cup)									
Kale, mustard greens or chard (1/2 cup)									
Spinach, cooked (1/2 cup)									
Spinach, raw as in salad (1 cup)									
Iceberg or head lettuce (1 cup)									
Romaine or leaf lettuce (1 cup)									
Celery (2–3 sticks)									
Peppers: green, yellow or red (3 slices)									
Onions as a garnish or in salad (1 slice)									
Onions as a cooked vegetable, rings or soup (1/2 cup)									

EGGS, MEAT, ETC.	Never, or less than once per month	1–3 per month	1 per week	2–4 per week	5–6 per week	1 per day	2–3 per day	4–5 per day	6+ per day
Eggs (1): Omega-3 fortified including yolk									
Eggs (1): Regular eggs including yolk									
Beef or pork hot dogs (1)									
Chicken or turkey hot dogs (1)									
Chicken/turkey sandwich or frozen dinner									
Other chicken or turkey, with skin (3 oz.)									
Other chicken or turkey, without skin (3 oz.)									
Bacon (2 slices)									

27. ***(continued)*** **For each food listed, fill in the circle indicating how often on average you have used the amount specified during the past year.**

EGGS, MEAT, ETC.	Never, or less than once per month	1–3 per month	1 per week	2–4 per week	5–6 per week	1 per day	2–3 per day	4–5 per day	6+ per day
Salami, bologna, or other processed meat sandwiches			W			D			
Other processed meats, e.g., sausage, kielbasa, etc. (2 oz. or 2 small links)			W			D			
Hamburger (1 patty) — Lean or extra lean			W			D			
Hamburger (1 patty) — Regular			W			D			
Beef, pork, or lamb as a sandwich or mixed dish, e.g., stew, casserole, lasagna, etc.			W			D			
Pork as a main dish, e.g., ham or chops (4–6 oz.)			W			D			
Beef or lamb as a main dish, e.g., steak, roast (4–6 oz.)			W			D			
Canned tuna fish (3–4 oz.)			W			D			
Breaded fish cakes, pieces, or fish sticks (1 serving, store bought)			W			D			
Shrimp, lobster, scallops as a main dish			W			D			
Dark meat fish, e.g., mackerel, salmon, sardines, bluefish, swordfish (3–5 oz.)			W			D			
Other fish, e.g., cod, haddock, halibut (3–5 oz.)			W			D			

BREADS, CEREALS, STARCHES	Never, or less than once per month	1–3 per month	1 per week	2–4 per week	5–6 per week	1 per day	2–3 per day	4–5 per day	6+ per day
Cold breakfast cereal (1 cup)			W			D			
Cooked oatmeal/cooked oat bran (1 cup)			W			D			
Other cooked breakfast cereal (1 cup)			W			D			
Bread (1 slice) — White bread, including pita			W			D			
Bread (1 slice) — Crispbreads (e.g., Wasa)			W			D			
Bread (1 slice) — Rye/Pumpernickel			W			D			
Bread (1 slice) — Whole wheat, oatmeal, other whole grain			W			D			
Crackers, regular or lowfat (6) e.g., Triscuits, Ritz			W			D			
Bagels, English muffins, or rolls (1)			W			D			
Muffins or biscuits (1)			W			D			
Pancakes or waffles (2 small pieces)			W			D			
Brown rice (1 cup)			W			D			
White rice (1 cup)			W			D			
Pasta, e.g., spaghetti, noodles, etc. (1 cup)			W			D			
Tortillas (1)			W			D			
French Fries (6 oz. or 1 serving)			W			D			
Potatoes, baked, boiled (1) or mashed (1 cup)			W			D			
Potato chips or corn/tortilla chips (small bag or 1 oz.)			W			D			
Pizza (2 slices)			W			D			

CARBONATED BEVERAGES

Consider the serving size as 1 glass, bottle or can for these carbonated beverages.

BEVERAGES	Never, or less than once per month	1–3 per month	1 per week	2–4 per week	5–6 per week	1 per day	2–3 per day	4–5 per day	6+ per day
Low-Calorie (sugar-free) types — Low-calorie beverage with caffeine, e.g., Diet Coke, Diet Mt. Dew			W			D			
Low-Calorie (sugar-free) types — Other low-cal bev. without caffeine, e.g., Diet 7-Up			W			D			
Regular types (not sugar-free) — Carbonated beverage with caffeine & sugar, e.g., Coke, Pepsi, Mt. Dew, Dr. Pepper			W			D			
Regular types (not sugar-free) — Other carbonated beverage with sugar, e.g., 7-Up, Root Beer, Ginger Ale			W			D			
OTHER BEVERAGES — Punch, lemonade, other non-carbonated fruit drinks, or sugared ice tea (1 glass, bottle, can)			W			D			
Beer, regular (1 glass, bottle, can)			W			D			
Light Beer, e.g., Bud Light (1 glass, bottle, can)			W			D			
Red wine (5 oz. glass)			W			D			
White wine (5 oz. glass)			W			D			
Liquor, e.g., vodka, gin, etc. (1 drink or shot)			W			D			
Plain water, bottled, sparkling, or tap (8 oz. cup)			W			D			
Herbal tea or decaffeinated tea (8 oz. cup)			W			D			
Tea with caffeine (8 oz. cup), including green tea			W			D			
Decaffeinated coffee (8 oz. cup)			W			D			
Coffee with caffeine (8 oz. cup)			W			D			

3/8" spine perf

27. ***(continued)*** **For each food listed, fill in the circle indicating how often on average you have used the amount specified during the past year.**

SWEETS, BAKED GOODS, MISCELLANEOUS		Never, or less than once per month	1–3 per month	1 per week	2–4 per week	5–6 per week	1 per day	2–3 per day	4–5 per day	6+ per day
Milk chocolate (bar or pack), e.g., Hershey's, M&M's				W			D			
Dark chocolate, e.g., Hershey's Dark or Dove Dark				W			D			
Candy bars, e.g., Snickers, Milky Way, Reeses				W			D			
Candy without chocolate (1 oz.)				W			D			
Cookies (1)	Fat free or reduced fat			W			D			
	Other ready made			W			D			
	Home baked			W			D			
Brownies (1)				W			D			
Doughnuts (1)				W			D			
Cake, homemade or ready made (slice)				W			D			
Pie, homemade or ready made (slice)				W			D			
Jams, jellies, preserves, syrup, or honey (1 Tbs)				W			D			
Peanut butter (1 Tbs)				W			D			
Popcorn (3 cups)	Fat free or light			W			D			
	Regular			W			D			
Sweet roll, coffee cake or other pastry (serving)	Fat free or reduced fat			W			D			
	Other ready made			W			D			
	Home baked			W			D			
Pretzels (1 small bag or serving)				W			D			
Peanuts (small packet or 1 oz.)				W			D			
Walnuts (1 oz.)				W			D			
Other nuts (small packet or 1 oz.)				W			D			
Oat bran, added to food (1 Tbs)				W			D			
Other bran, added to food (1 Tbs)				W			D			
Chowder or cream soup (1 cup)				W			D			
Ketchup or red chili sauce (1 Tbs)				W			D			
Splenda (1 packet)				W			D			
Other artificial sweetener (1 packet)				W			D			
Olive oil added to food or bread (1 Tbs)				W			D			
Low-fat or fat-free mayonnaise (1 Tbs)				W			D			
Regular mayonnaise (1 Tbs)				W			D			
Salad dressing (1-2 Tbs)				W			D			

Type of salad dressing: ○ Nonfat ○ Low-fat ○ Olive oil ○ Other vegetable oil

28. Liver: beef, calf or pork (4 oz.) ○ Never ○ Less than 1/mo ○ 1/mo ○ 2–3/mo ○ 1/week or more

Liver: chicken or turkey (1 oz.) ○ Never ○ Less than 1/mo ○ 1/mo ○ 2–3/mo ○ 1/week or more

29. How much of the visible fat on your beef, pork or lamb do you remove before eating?
○ Remove all visible fat ○ Remove most ○ Remove small part of fat ○ Remove none ○ Don't eat meat

30. How often do you eat fried or sautéed food at home? (Exclude "Pam"-type spray)
○ Less than once a week ○ 1–3 times per week ○ 4–6 times per week ○ Daily

31. What kind of fat is usually used for frying and sautéing at home? (Exclude "Pam"-type spray)
○ Real butter ○ Margarine ○ Vegetable oil ○ Vegetable shortening ○ Lard ○ N/A

32. What kind of fat is usually used for baking at home?
○ Real butter ○ Margarine ○ Vegetable oil ○ Vegetable shortening ○ Lard ○ N/A

33. What type of cooking oil is usually used at home? (e.g., Mazola Corn Oil) Specify brand and type → ______

34. How often do you eat deep fried chicken, fish, shrimp, clams or onion rings away from home?
○ Less than once a week ○ 1–3 times per week ○ 4–6 times per week ○ Daily

35. How often do you eat toasted breads, bagel or English muffin (e.g., slice or 1 half bagel)?
○ Less than once a week ○ 1–3 times per week ○ 4–6 times per week ○ Daily ○ 2+ times/day

36. Are there any other important foods that you usually eat at least once per week?

Include for example: Applesauce, mushrooms, bulgur, couscous, radish, horseradish, Eggbeaters, dates, figs, mango, mixed dried fruit, papaya, wheat germ, custard, venison, hot peppers, pickles, olives, SlimFast, Ensure (regular, plus or light), Power/Sports bars.

(Do not include dry spices and do not list something that has been listed in the previous sections.)

	Other foods that you usually eat at least once per week	Servings per week
(a)		
(b)		
(c)		

Thank you! Please return forms in prepaid return envelope to: Dr. Walter Willett, 677 Huntington Avenue, Boston, MA 02115.
