# Supplementary material for: A Prospective Study of Alcohol Consumption and Smoking and the Risk of Major Gastrointestinal Bleeding in Men
Source: PLoS One. 2016 Nov 8;11(11):e0165278. doi: 10.1371/journal.pone.0165278 (PMC5100927; doi:10.1371/journal.pone.0165278)
Supplement: S1 Table — (DOCX) [file pone.0165278.s002.docx]

**S1 Table.** **Multivariable RRs and 95% CIs of GI bleeding according to alcohol intake and use of nonsteroidal anti-inflammatory drugs^a^**

|  | **Alcohol intake (g/day)** | | |
| --- | --- | --- | --- |
|  | **0** | **1-14** | **≥15** |
| **All GI bleeding^b^** |  |  |  |
| No NSAIDS/Aspirin | 1.0 | 0.86 (0.43, 1.73) | 1.44 (0.68, 3.06) |
| Yes NSAID/Aspirin^c^ | 1.0 | 1.37 (0.85, 2.19) | 1.75 (1.07, 2.88) |
| **Upper GI bleeding^d^** |  |  |  |
| No NSAIDS/Aspirin | 1.0 | 1.01 (0.33, 3.10) | 1.57 (0.47, 5.21) |
| Yes NSAID/Aspirin | 1.0 | 1.16 (0.61, 2.23) | 1.75 (0.88, 3.45) |
| **Lower GI bleeding^d^** |  |  |  |
| No NSAIDS/Aspirin | 1.0 | 0.87 (0.34, 2.23) | 1.11 (0.37, 3.33) |
| Yes NSAID/Aspirin | 1.0 | 1.61 (0.73, 3.54) | 1.60 (0.69, 3.70) |

^a^ Multivariable RR adjusted for age in years, study period in 4-year intervals, smoking (past/current),

body mass index (<21, 25-29, 30-31, ≥32 kg/m^2^), physical activity (quintiles) using cumulative updating

to examine alcohol consumption

^b^ Includes 28 cases of bleeding of unknown etiology and 9 cases of small bowel bleeding

^c^ Use of NSAIDs/aspirin defined as use at least 2 times per week of either NSAIDs and/or aspirin.

^d^ Upper GI bleeding was defined as bleeding originating from the esophagus, stomach, or duodenum;

lower GI bleeding was defined as bleeding arising from the colon or rectum.
