# Supplementary material for: A Prospective Study of Alcohol Consumption and Smoking and the Risk of Major Gastrointestinal Bleeding in Men
Source: PLoS One. 2016 Nov 8;11(11):e0165278. doi: 10.1371/journal.pone.0165278 (PMC5100927; doi:10.1371/journal.pone.0165278)
Supplement: S2 Table — (DOCX) [file pone.0165278.s003.docx]

**S2 Table.** **Alcohol Consumption and Risk of Major GI Bleeding According to Bleeding Source**

|  | **0** | **1-4** | **5-14** | **≥15** | ***p* value for linear trend**^a^ |
| --- | --- | --- | --- | --- | --- |
| Person-years | 151156 | 244945 | 260621 | 227074 |  |
| **Ulcer bleeding** |  |  |  |  |  |
| No. of cases | 12 | 22 | 33 | 37 |  |
| Age-adjusted HR (95% CI)^b^ | 1.0 | 0.93 (0.46, 1.87) | 1.28 (0.66, 2.49) | 1.59 (0.83, 3.05) | 0.037 |
| Multivariable1 HR (95% CI)^c^ | 1.0 | 0.90 (0.44, 1.83) | 1.31 (0.67, 2.56) | 1.61 (0.82, 3.14) | 0.034 |
| Multivariable 2 HR (95% CI)^d^ | 1.0 | 0.90 (0.44, 1.83) | 1.30 (0.66, 2.54) | 1.60 (0.82, 3.12) | 0.037 |
| **Inflammatory bleeding^e^** |  |  |  |  |  |
| No. of cases | 3 | 13 | 11 | 16 |  |
| Age-adjusted HR (95% CI)^b^ | 1.0 | 2.28 (0.65, 8.01) | 1.78 (0.50, 6.39) | 3.01 (0.88, 10.4) | 0.125 |
| Multivariable 1 HR (95% CI)^c^ | 1.0 | 2.05 (0.58, 7.23) | 1.49 (0.41, 5.42) | 2.42 (0.69, 8.53) | 0.272 |
| Multivariable 2 HR (95% CI)^d^ | 1.0 | 2.08 (0.59, 7.35) | 1.48 (0.41, 5.40) | 2.38 (0.67, 8.38) | 0.271 |
| **Diverticular bleeding^f^** |  |  |  |  |  |
| No. of cases | 12 | 25 | 28 | 23 |  |
| Age-adjusted HR (95% CI)^b^ | 1.0 | 1.05 (0.53, 2.09) | 1.06 (0.54, 2.10) | 0.96 (0.48, 1.94) | 0.770 |
| Multivariable 1 HR (95% CI)^c^ | 1.0 | 1.02 (0.51, 2.04) | 1.04 (0.53, 2.07) | 0.93 (0.45, 1.90) | 0.722 |
| Multivariable 2 HR (95% CI)^d^ | 1.0 | 0.98 (0.49, 1.96) | 1.02 (0.51, 2.03) | 0.90 (0.44, 1.85) | 0.714 |

^a^ *p* value for trend calculated using the median value in each category of alcohol consumption as a continuous variable in the regression models

^b^ Adjusted for age in years and study period in 4-year intervals; using cumulative updating to examine alcohol consumption

^c^ Adjusted for smoking (past/current), body mass index (<21, 25-29, 30-31, ≥32 kg/m^2^), physical activity (quintiles), regular use of aspirin (at least 2 times per week; yes/no), regular use of NSAIDs (at least 2 times per week; yes/no); using cumulative updating to examine alcohol consumption

^d^ Adjusted for multivariate model 1 plus medication use (proton pump inhibitors, H2 receptor antagonists, selective serotonin reuptake inhibitors, warfarin and/or clopidogrel) and comorbid disease (myocardial infarction, stroke or transient ischemic attack, rheumatoid arthritis, degenerative joint disease, peripheral vascular disease, chronic obstructive pulmonary disease, chronic kidney disease).

^e^ Inflammatory bleeding was defined as bleeding from esophagitis, gastritis or duodenitis.

^f^ Diverticular bleeding was defined if stigmata of recent hemorrhage or active diverticular bleeding was identified or if no other source of lower GI bleeding was identified (presumed diverticular bleeding).
